# Impact of the COVID-19 epidemic at a high-volume facility in gynecological oncology in Tokyo, Japan: a single-center experience

**DOI:** 10.1186/s13048-020-00711-x

**Published:** 2020-09-11

**Authors:** Yuya Nogami, Yusuke Kobayashi, Kosuke Tsuji, Megumi Yokota, Hiroshi Nishio, Masaru Nakamura, Wataru Yamagami, Tohru Morisada, Eiichiro Tominaga, Kouji Banno, Daisuke Aoki

**Affiliations:** Department of Obstetrics & Gynecology, Keio University School of Medicine, 35 Shinanomachi, Shinjuku-ku, 160-8582 Tokyo, Japan

**Keywords:** Ovarian cancer, Ovarian tumor, Cervical cancer, COVID-19, SARS-CoV-2, Universal screening, RT-PCR, CT

## Abstract

**Background:**

The number of cases of novel coronavirus disease 2019 (COVID-19) in Japan have risen since the first case was reported on January 24, 2020, and 6225 infections have been reported as of June 30, 2020. On April 8, 2020, our hospital began screening patients via pre-admission reverse transcriptase-polymerase chain reaction (RT-PCR) for severe acute respiratory syndrome coronavirus 2 (SARS-CoV-2) and chest computed tomography (CT). Although no patients exhibited apparent pneumonia, treatment delay or changes in treatment plans were required for a few patients based on the results of screening tests. During an emerging infectious disease pandemic, the likelihood of being infected, as well as the disease itself, affects clinical decision making in several ways. We summarized and presented our experience.

**Case presentation:**

After the introduction of pre-admission screening, RT-PCR and CT were performed in 200 and 76 patients, respectively, as of June 30, 2020. The treatment of five patients, including two patients with cervical cancer, two patients with ovarian tumors, and one patient with ovarian cancer, was affected by the results. Two asymptomatic RT-PCR-positive patients did not develop COVID-19, but their treatment was delayed until the confirmation of negative results. The other three patients were RT-PCR-negative, but abnormal CT findings suggested the possibility of COVID-19, which delayed treatment. The patients receiving first-line preoperative chemotherapy for ovarian cancer had clinically evident exacerbations because of the treatment delay.

**Conclusion:**

During the epidemic phase of an emerging infectious disease, we found that COVID-19 has several other effects besides its incidence. The postponing treatment was the most common, therefore, treatment of ovarian tumors and ovarian cancer was considered to be the most likely to be affected among gynecological diseases. Protocols that allow for easy over-diagnosis can be disadvantageous, mainly because of treatment delays, and therefore, the protocols must be developed in light of the local infection situation.

## Background

Since the first case of novel coronavirus disease 2019 (COVID-19) was reported in Tokyo on January 24, 2020 [1], the number of cases has continued to rise [2]. “A state of emergency” was declared from April 7 to May 25, 2020. The total number of cases exceeded 5000 on May 15, 2020.

As of June 30, 2020, 6225 infections have been reported in Tokyo, which has a population of approximately 20 million people [2]. Our hospital is located in the center of Tokyo, and it focuses on the treatment of gynecological malignancies, including ovarian cancer. We perform approximately 1200 surgeries a year, of which approximately 550 are surgeries for malignancy. At the end of March 2020, an outbreak of nosocomial infections among junior residents occurred, but no nosocomial infections occurred among gynecological inpatients. On April 8, 2020, the hospital began screening via pre-admission reverse transcriptase-polymerase chain reaction (RT-PCR) for severe acute respiratory syndrome coronavirus 2 (SARS-CoV-2) in-house. Chest computed tomography (CT) was also performed in scheduled surgical cases with general anesthesia. Patients requiring emergency admission were managed in private rooms, and after the possibility of COVID-19 was eliminated, including negative RT-PCR results, isolation management was halted. Patients with related symptoms such as fever, cough, and taste disorder at the time of emergency admission also underwent chest CT.

Two asymptomatic RT-PCR-positive patients were receiving treatment for gynecological malignancies. One patient with cervical cancer was followed up in the isolation ward for inpatient management, and the other patient with ovarian cancer was followed up at home. Both patients were confirmed to be RT-PCR-negative without any disease development. Although none of the patients exhibited apparent pneumonia, a few cases, including ovarian tumors or cancers, of postponed or changed treatment were prompted by the screening tests.

During an emerging infectious disease pandemic, the likelihood of being infected, as well as the disease itself, affects clinical decision making in a variety of ways. Especially, treatment strategies for ovarian tumors and cancers seemed to be more susceptible to the impact of the COVID-19 epidemic among gynecological diseases. We have summarized and presented our experience.

## Case presentation

After the introduction of RT-PCR as a pre-admission screening modality in the gynecology department, RT-PCR and CT were performed in 200 and 76 patients, respectively, and 20 patients who admitted emergently required RT-PCR for release from isolation, as of June 30, 2020.

Only two patients were RT-PCR-positive as mentioned previously, but a few patients had abnormal findings on chest CT. The sensitivity of RT-PCR is reported as 70%, and the elimination of COVID-19 as a possible diagnosis was difficult at that time; consequently, subsequent treatment was affected.

### Case 1: 49-year-old woman, asymptomatic RT-PCR-positive

The patient had stage IIIB squamous cell carcinoma of the cervix. Recurrence involving para-aortic lymph node metastasis and peritoneal dissemination was noted 9 months after concurrent chemoradiotherapy (CCRT). She was planned to participate in a randomized clinical trial of immune check point inhibitor with cytotoxic drugs for the recurrent disease. During pre-hospitalization screening for her first scheduled treatment, she tested positive for COVID-19 via RT-PCR. She was asymptomatic, and CT revealed no abnormal findings in the lung fields, however, left supraclavicular lymph node enlargement was observed. She was admitted to the hospital for isolation and observation for COVID-19. Repeat RT-PCR was negative on the sixth day, and the patient was discharged the following day after RT-PCR was again negative. There was no provision in the protocol of the clinical trial for COVID-19, but pre-registration tests of the trial was expired due to her hospitalization. Thus, the patient re-consented to the trail and was required to have pre-registration tests again. We started her treatment one month behind the initial schedule.

### Case 2: 49-year-old woman, asymptomatic RT-PCR-positive

The patient was scheduled for surgery for an ovarian tumor. Magnetic resonance imaging (MRI) revealed a 9-cm ovarian tumor with a solid component, suggesting the possibility of borderline malignancy or worse. Unilateral adnexal resection via laparoscopy was planned to prevent rupture/torsion and facilitate the pathological diagnosis. Preoperative RT-PCR was positive, whereas CT revealed no abnormalities. The patient was asymptomatic, and she was isolated at home for observation (the political policy has changed from the aforementioned example, permitting follow-up at home or in a hotel). Subsequently, the patient was confirmed negative via RT-PCR after 1 and 2 weeks, and surgery was planned again. The final pathological diagnosis was a benign lesion, but surgery was delayed by 4 weeks.

### Case 3: 50-year-old woman

**Fig. 1:**
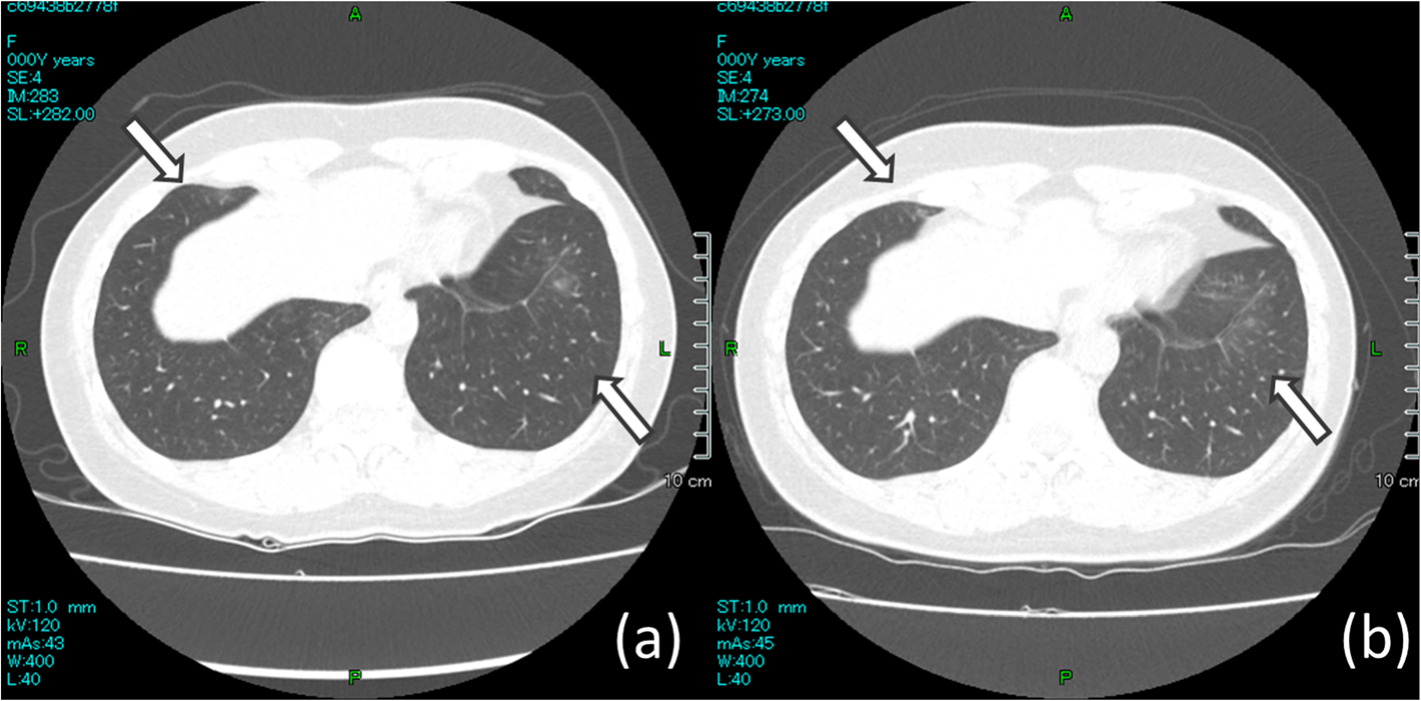
Computed tomography (CT) images of the lung field of Case 3. At the time of initial presentation (**a**) and 3 weeks later (**b**). CT revealed ground-glass opacity in the bilateral inferior lung fields (arrows) but no change over time

The patient was scheduled for surgery for an ovarian tumor. MRI identified a 5-cm ovarian enlargement that was diagnosed as mucinous cyst adenoma. Surgery was planned to prevent torsion and diagnose the pathology. The patient was asymptomatic and RT-PCR-negative, but CT uncovered ground-glass opacity (GGO) in the bilateral inferior lung fields (Fig. 1a). The results, although non-specific, were consistent with early COVID-19, and the patient was retested after 2 weeks. During the observation, the patient progressed without any onset of disease, and CT was repeated after 2 and 3 weeks, with no change in GGO (Fig. 1b). Judging from the patient’s history, the possibility of COVID-19 was eliminated, and a diagnosis of stale inflammatory changes was made. The original treatment plan was delayed by 3 weeks.

### Case 4: 51-year-old woman (this case was reported before as a preliminary report [3])

**Fig. 2:**
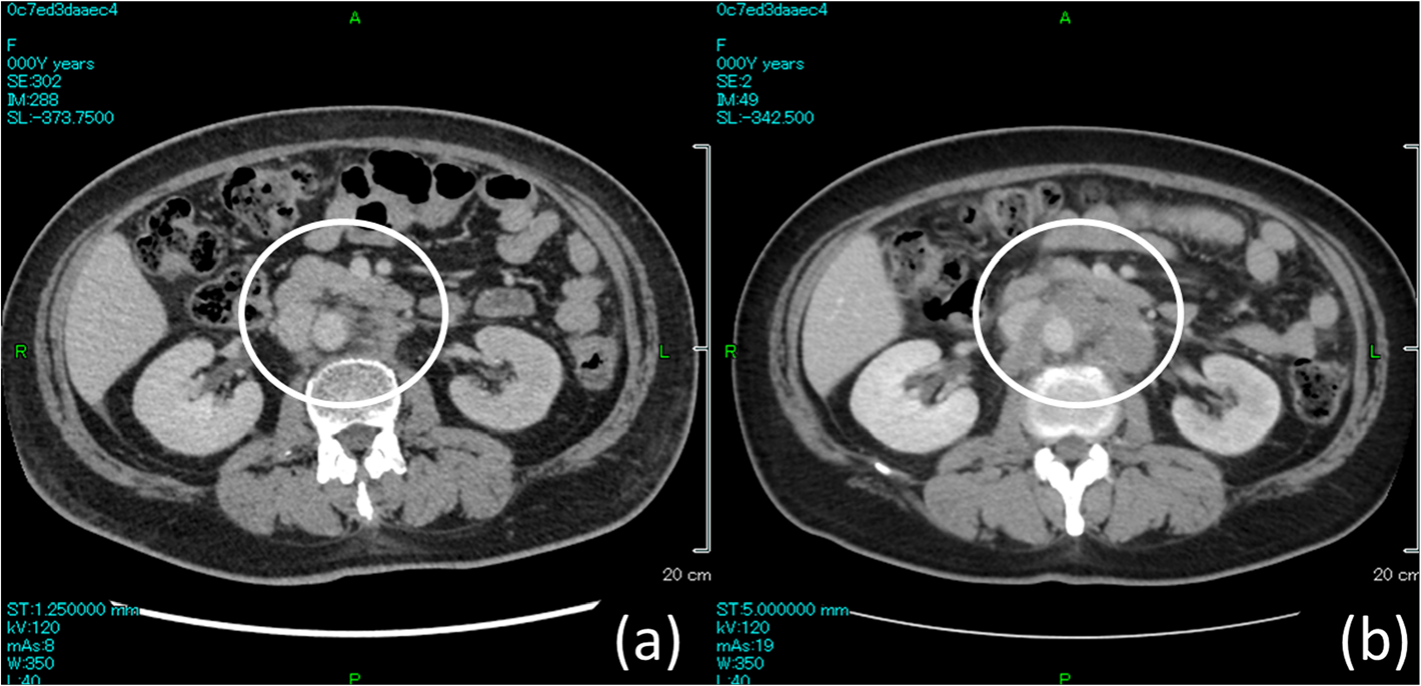
Abdominal computed tomography (CT) images of Case 4. The metastatic para-aortic lymph nodes (in circles) had shrunk after three cycles of chemotherapy (**a**), but re-growth occurred during the delay in treatment (**b**)

**Fig. 3:**
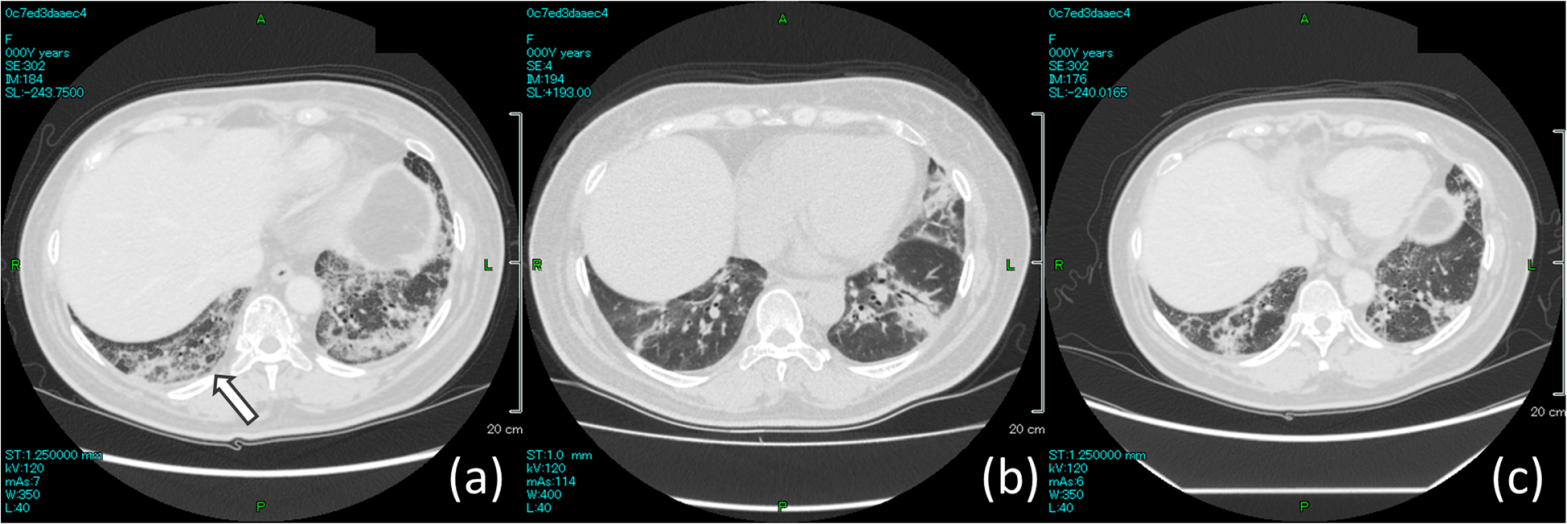
Computed tomography (CT) images of the lung field of Case 4. CT revealed the new appearance of ground-glass opacity, infiltrative shadows, and a rounded morphology (arrow) in the bilateral middle and lower lung fields (**a**). Fibrosis developed within 2 weeks (**b**) and then tended to disappear (**c**). The findings were highly suspicious of coronavirus disease 2019. Figure 3a was reprinted from reference [3]

The patient had stage IIIC serous carcinoma of the ovaries complicated by deep vein thrombosis and diabetes mellitus. She presented to the clinic with a chief complaint of abdominal distension, and a thorough examination revealed the aforementioned diagnosis. On pretreatment examination, deep venous thrombus was noted, and ascitic fluid retention was evident, which led to the use of neoadjuvant chemotherapy. A tri-weekly regimen of paclitaxel 180 mg/m^2^, carboplatin AUC 5, and bevacizumab 15 mg/kg was started. CT at the end of three cycles revealed shrinkage of the primary tumor and retroperitoneal lymph node metastasis (Fig. 2a), a reduction in peritoneal disseminated lesions, and a decrease in ascites; however, the new appearance of GGO, infiltrative shadows, and a rounded morphology in the bilateral middle and lower lung fields were noted (Fig. 3a). The CT findings were highly suspicious of COVID-19, but RT-PCR returned negative results. Chemotherapy was temporarily discontinued, and 2 weeks later, CT was repeated. Infiltrative shadows with fibrosis were observed in both inferior lung fields, which was consistent with the resolution of pneumonia (Fig. 3b). Repeat CT was performed 2 weeks later, and the GGO tended to dissipate (Fig. 3c). However, swelling of the retroperitoneal lymph nodes had returned. Repeat CT 2 weeks later confirmed no change in the pneumonia. Ascites had worsened, and the retroperitoneal lymph nodes were larger (Fig. 2b). RT-PCR was performed, and the results were again negative. The possible causes of pneumonia were COVID-19 and drug-induced pneumonia. However, even if COVID-19 was present, the disease was not active, and it was determined that chemotherapy would take priority because of exacerbation of the primary disease. Considering the possibility of drug-induced pneumonia, the drug was changed from paclitaxel to liposomal doxorubicin, and chemotherapy was resumed. Treatment was delayed by 7 weeks in this case, and the delay resulted in a clinically evident exacerbation of the case.

### Case 5: 58-year-old woman

The patient had stage IIIB adenosquamous carcinoma of the cervix, and recurrence with re-growth of the primary tumor was noted 2 months after CCRT. Because of tumor-induced hydronephrosis, bilateral ureteral stents were indwelled.

**Fig. 4:**
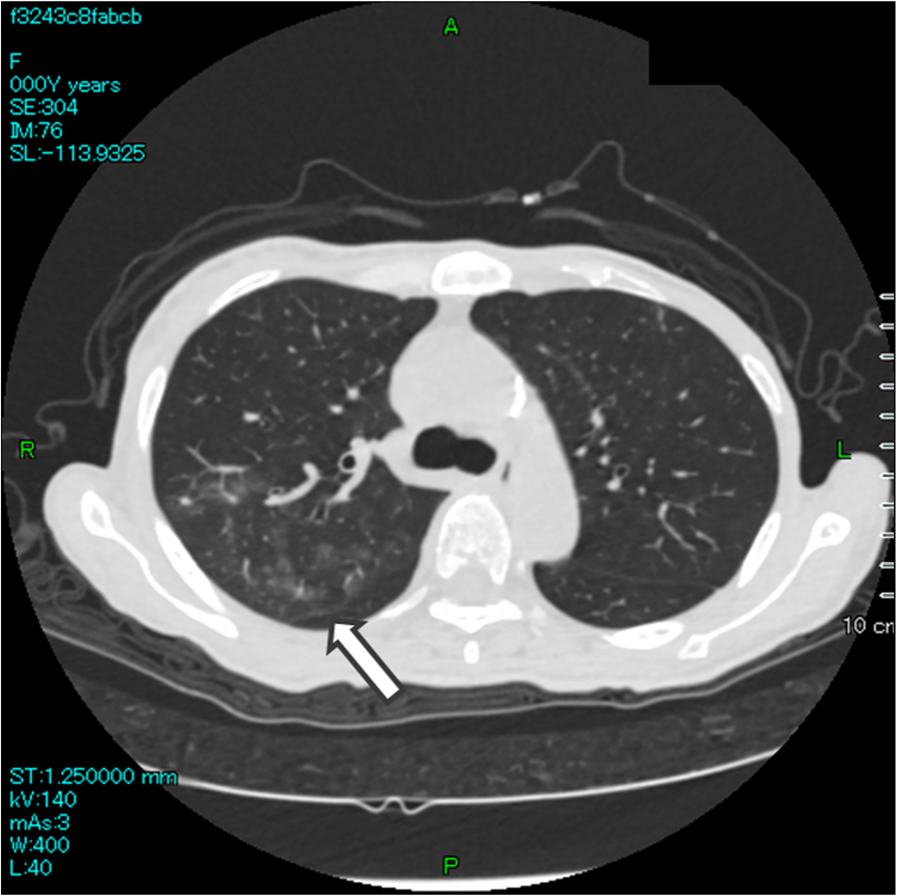
Computed tomography (CT) images of the lung field of Case 5. CT revealed ground-glass opacity and granular shadows in the right upper lobe of the lung field (arrow)

She was admitted to the hospital on an emergency basis because of back pain and deterioration of kidney function. Multiple bone metastases and pathological fractures were noted on CT on admission, but simultaneously, GGO and a granular shadow were noted in the right upper lobe of the lung field, suggesting the possibility of COVID-19 (Fig. 4). RT-PCR was performed the next day, and the results were negative. There were no related symptoms such as fever or respiratory symptoms other than pain. On the fourth day of hospitalization, RT-PCR was repeated with negative results, and the patient was released from isolation to a private room. Bone metastases were irradiated, and the patient’s pain was controlled with opioids. In addition, urinary tract obstruction and infection were treated, the patient’s renal function improved, and she was discharged after 1 month of hospitalization. Before discharge, the patient was re-examined via CT, and although GGO in the upper lung field was exacerbated, the likelihood of morbidity was low. Therefore, the patient was considered to have other conditions such as atypical pneumonia or drug-induced pneumonia. The situation required inpatient management, regardless of the suspected COVID-19, to control other symptoms and conditions. Although the only medical issue was the need for isolation for infection control during hospitalization, it was possible that a suspected infection during the end-of-life care may have caused emotional distress. In fact, the patient was able to stay at home for 2 weeks, she was later re-admitted to the hospital, at which she died of sepsis. If the suspicion of COVID-19 was not resolved, the quality of life during end-of-life case could have been diminished because of the lack of time with family and adequate palliative care.

## Discussion and conclusion

### Difficulty in deciding whether to postpone treatment

The global spread of COVID-19, an emerging infectious disease, has exerted a variety of effects on high-volume gynecological oncology practices, and these effects were attributable to both the disease itself and the suspicion of disease. In most cases, the mere postponement of a necessary scheduled treatment may have a significant impact, but in the case of cancer treatment, the impact of the treatment delay represents a therapeutic disadvantage.

In the case of surgery, it is desirable to conduct the operation as soon as possible before the disease progresses. In the case of chemotherapy, if treatment is discontinued temporarily, then the dose intensity will be reduced. For instance, for six cycles of tri-weekly paclitaxel and carboplatin therapy, if COVID-19 causes a 4-week delay during treatment, the relative dose intensity will be approximately 82%. The relationship between the relative dose intensity and prognosis has been described for various cancer types [4–9]. Even if treatment is deferred, it is not desirable for treatment to be postponed unnecessarily. In addition to the physical therapeutic disadvantages, delays in planned treatment were reported to cause anxiety and depression for patients, leading to lower quality of life [10].

Conversely, patients with cancer are especially likely to have a high risk of severe COVID-19 [11, 12]. In addition, surgery in patients with COVID-19, even in those without coincident cancer, may result in miserable outcomes [13–15]. Because of the limited sensitivity of RT-PCR, treatment decisions must be made with caution.

The recommendation for suspension of chemotherapy in patients with COVID-19 is considered reasonable, and a delay of 2–4 weeks is currently recommended [16]. A patient with ovarian cancer was reported to have safely resumed chemotherapy in COVID-19 convalescence with a similar withdrawal period [17]. However, it is unclear whether the required duration of treatment interruption is similar between patients with apparent pneumonia and asymptomatic RT-PCR-positive patients. There was a report of a patient who tested positive antibodies during chemotherapy. Although it was unclear at what point she contracted, the patient was able to continue chemotherapy safely [18].

### Decision based on CT findings

Regarding imaging, typical CT findings of COVID-19 are said to be peripheral, inferior, and bilateral, starting with bilateral peripheral frosted shadows in the early stages and extending to tuck the lobes of the lungs during disease progression. However, it has been reported that more than half of patients have normal findings in the early stages of the disease [19, 20]. Thus, normal findings on CT do not eliminate the possibility of COVID-19 in patients with a suspicious history and symptoms.

Meanwhile, a meta-analysis uncovered a specificity of 37% for CT [21]. If there are only abnormalities in the CT findings, different diagnoses are more likely. Because little remains known about COVID-19, we have concerns regarding the worst consequences of forcing treatment. Because of this cautiousness, treatment is often delayed, and judgment is withheld until follow-up and repeat RT-PCR confirm the absence of COVID-19.

### Susceptibility of the treatment strategies for ovarian tumors and ovarian cancer

Given the above two discussions, it would be difficult to diagnose accurately during an epidemic. When we allowed some over-diagnosis, there would be more cases where treatment is discontinued. This would most likely affect the treatment of ovarian tumors and ovarian cancer (including fallopian tube and peritoneal cancers). Three of the five cases in the present study were ovarian tumors and ovarian cancer.

The first reason is that in many ovarian tumors, the surgeries are required for pathological diagnosis. Once a diagnosis of malignancy is made in an outpatient examination, such as cervical or uterine cancer, the priority of treatment would be increased. In the worst-case scenario, such as surgical restrictions or hospital closures, the patient could be transferred to another gynecological malignancy treatment facility. Patients who are not suspected of having aggressive malignant findings on imaging may be treated as benign and put off. Case 2 and 3 in this series had resulted in benign histology, but if a malignant, significant delay in treatment.

The second reason is that in advanced cases of ovarian cancer, the treatment plan of neoadjuvant chemotherapy (NAC) followed by interval debulking surgery (IDS) is more often chosen than cervical or uterine cancer. IDS requires careful planning in advance, such as anticipating the duration of bone marrow recovery from the latest chemotherapy, but deferral due to COVID-19 could suddenly ruin that plan. In case 4, the patient was suspected of having COVID-19, and while the chemotherapy was postponed, the patient showed worsening of the disease and did not reach IDS.

This issue had been discussed, not just from our own experiences. Guidelines and recommendations have been issued by various organizations, including countries with more widespread conditions, such as China, Italy, and the United States [22, 23]. According to these guidelines, CCRT for cervical cancer was recommended with high priority, and many patients could receive standard treatment. For advanced ovarian cancer, there were recommendations to avoid highly invasive primary debulking surgery, which is likely to occupy the intensive care unit. Thus, a strategy of neoadjuvant chemotherapy to interval debulking surgery would be recommended, but this, of course, needs to be balanced with individual patient prognosis, which is a difficult decision [24, 25]. Surgery for endometrial cancer was also considered a low priority for early-stage cancer [23]. Ovarian cancer is the most susceptible among the three major gynecological cancers, considering the prognoses and the rate of advanced cancer [26].

### Preventive measures for patients with cancer during hospital closures caused by nosocomial infections

In response to the spread of epidemics in Japan, the Japanese Society of Obstetrics and Gynecology has created a network system, named Perinatal Early Assessment and Communication system for Emergencies, for sharing information about hospital functioning in real-time for hospitals across the country [27]. This system was established for disaster situations, but it is intended for perinatal care only. There is no similar system for the treatment of gynecological malignancies, and if most hospitals are shut down because of the nosocomial spread of COVID-19, many patients may be unable to obtain treatment. In fact, because of the COVID-19 epidemic in Tokyo, other high-volume gynecological malignancy treatment facilities in the city have temporarily limited medical functions [28]. We have established an information-sharing network at 19 hospitals in Tokyo (Gynecologic oncological surgery communication in Tokyo in New Infection pandemic by Coronavirus; URL undisclosed). As of June 30, 2020, although the epidemic has not yet resulted in the transfer of patients to other hospitals en masse, we are preparing for a large surge in the number of patients seeking treatment.

### Sensitivity, specificity, benefits, and drawbacks of universal testing based on the epidemic situation

Because nosocomial infections were identified among junior clinical residents of our hospital staff [29], the executive committee, as a prudent response, implemented a screening system using RT-PCR and CT for all patients before admission, resulting in possible over-diagnosis. As a result, we found that approximately 6% of asymptomatic individuals were RT-PCR-positive in the week of April 13–19, 2020 [30]. Based on previous reports, the sensitivity and specificity of RT-PCR were 70 and 100%, respectively [31], and those of CT are 94 and 37% [21]. Thus, in a population that is 6% RT-PCR-positive, the positive predictive value and negative predictive value of RT-PCR are 100 and 97.3%, respectively, and those of CT are 12.3 and 98.5%, respectively. Indeed, a screening program is likely to be a solid barrier to preventing the admission of unrecognized infected people. However, because a positive CT finding alone requires treatment delay and follow-up, approximately 90% of patients with a positive CT finding may experience an unnecessary delay in treatment. Testing should be selected on the basis of the local infection situation. Because the emergency declaration was lifted and the infection rate in the general population was believed to have decreased further, preoperative screening using CT was discontinued on June 22, 2020.

To date, the factors that predict severe disease for asymptomatic and mildly ill individuals have been identified [32], but it is hoped that investigation concerning the presence or absence of true infection for asymptomatic individuals will continue [31].

During the epidemic phase of an emerging infectious disease, we found that the disease has several other effects besides its incidence. The postponing treatment was the most common, therefore, treatment of ovarian tumors and ovarian cancer was considered to be the most likely to be affected among gynecological diseases. Protocols that allow for easy over-diagnosis can be disadvantageous, mainly because of to treatment delays, and therefore, protocols must be developed in light of the local infection situation.

## Data Availability

All data used during this study are included in this published article.

## Abbreviations

CCRT: Concurrent chemoradiotherapy
GGO: Ground-glass opacity
